# Sulfonylurea-Induced Hypoglycemia in a Patient With Cirrhosis

**DOI:** 10.7759/cureus.8513

**Published:** 2020-06-08

**Authors:** Corey Braet, Yasmine Hussein Agha, Ali Taleb, Charles Buess, John Millard

**Affiliations:** 1 Internal Medicine, University of Kansas School of Medicine-Wichita, Wichita, USA; 2 Pharmacy, Robert J. Dole Veterans Affairs Medical Center, Wichita, USA

**Keywords:** sulfonylurea compounds, liver cirrhosis, drug contraindications

## Abstract

Type 2 diabetes mellitus is highly prevalent among patients with cirrhosis. The pharmacological management of this disease in patients with chronic liver disease remains controversial, however. Insulin secretagogues such as sulfonylureas are associated with a high risk of hypoglycemia among diabetics. In patients with cirrhosis, this risk is more pronounced due to decreased hepatic clearance, concurrent alcoholism, hypoalbuminemia, and acute liver decompensation. In this case report, we present a case of severe refractory hypoglycemia secondary to glipizide in a patient with alcoholic cirrhosis. We believe that the use of sulfonylureas in this patient population should be contraindicated to avoid debilitating neurologic damage and death following hypoglycemia.

## Introduction

Type 2 diabetes mellitus (DM) is highly associated with chronic liver disease, with an overall prevalence estimated at 31% among patients with cirrhosis [1]. Treating DM with sulfonylureas (SUs) in patients with cirrhosis is challenging since these drugs are metabolized by the liver. Most guidelines consider SUs safe in this patient population, whereas others recommend dose reduction [2,3]. The extent of liver damage in patients with cirrhosis is unpredictable and difficult to quantify, whether the patient is in a compensated state or deteriorating. With that in mind, the risk of hypoglycemia is expected to be higher in patients with cirrhosis taking SUs compared with diabetics without liver disease taking SUs. Despite the debilitating and fatal outcomes of hypoglycemia, no guidelines have discussed contraindicating SUs in patients with chronic liver disease. We present a case of severe refractory hypoglycemia secondary to glipizide in a patient with alcoholic cirrhosis.

## Case presentation

A 69-year-old male with a history of alcoholic liver cirrhosis and DM presented to the emergency department with weakness since awakening that morning. He was conscious but lethargic and was not oriented to time or place. Neurological examination was unremarkable for focal neurological deficits other than mild slurring of his speech. He denied having any seizure activity or loss of consciousness prior to arrival. His last alcoholic drink was three days prior to presentation. On physical examination, he had icterus, was mildly jaundiced, and appeared malnourished. He had minimal ascites and mild bilateral non-pitting lower limb edema. His lungs were clear, heart sounds were normal, and abdomen was non-tender.

He was afebrile, tachycardic with a heart rate of 120 beats per minute, and normotensive. Serum glucose was measured at bedside and was profoundly low (40 mg/dL). He was initially managed with multiple boluses of 50% dextrose, glucose gel every 15 minutes, and a continuous infusion of 10% dextrose in water in a stepwise manner. However, his blood sugar failed to stabilize. Initial laboratory work-up revealed leukocytosis, thrombocytopenia, acute kidney injury, hypoalbuminemia, transaminitis, and hyperbilirubinemia (Table 1). His serum ethanol was undetectable, and urine drug screen was negative. Urine SU screen was ordered as well and was still pending on the first day of admission. His MELD-Na score (Model for End-stage Liver Disease with Sodium) was 25, which translated to a 15% chance of mortality within three months. His liver function test and MELD-Na score obtained six months prior to admission were similar to his current results.

**Table 1:** Initial laboratory work-up

Laboratory tests	Results	Reference range
White blood cell count	18.9	4.8–10.8 x 10^3^/µL
Hemoglobin	12	12–16 gm/dL
Platelets	100	150–400 x 10^3^/µL
Sodium	135	136–145 meq/L
Potassium	4.5	3.5–5.1 meq/L
Chloride	102	96–106 meq/L
Bicarbonate	26	23–29 meq/L
Blood urea nitrogen	28	6–20 mg/dL
Creatinine	1.85	0.6–1.3 mg/dL
Albumin	1.5	3.5–5.5 g/dL
Aspartate aminotransferase	152	10–40 U/L
Alanine aminotransferase	86	10–40 U/L
Alkaline phosphatase	95	30–120 U/L
Total bilirubin	5.5	0.3–1.0 mg/dL
Unconjugated bilirubin	4	0.2–0.7 mg/dL
International normalized ratio	1.5	0.8–1.1
Ammonia	30	40–70 μg/dL
Ethanol	< 5	˂5 mg/dL

Computed tomography (CT) of the brain without contrast did not show any acute abnormalities. Upon review of his medication list, it was noted that he was on glipizide for the treatment of DM, and his last dose was the night prior to presentation. He had been on this medication for years and had never developed hypoglycemia. Since SU toxicity was the most probable cause of his persistent hypoglycemia, he was given 50 mcg of octreotide subcutaneously every eight hours in addition to a continuous infusion of 5% dextrose in half normal saline until serum glucose increased and was maintained within the normal range. Three days later, his urine SU screen showed a urine glipizide level of 480 ng/mL (reference: <5 ng/mL).

## Discussion

Glucose intolerance and DM are highly prevalent among patients with cirrhosis [4]. Studies have shown that glucose intolerance is found in 80% of patients with cirrhosis, whereas the prevalence of overt DM was found to range from 10% to 30% [4,5]. This has been reported to be caused by increased insulin resistance in patients with cirrhosis [2]. This association is even more pronounced in patients with alcoholic liver disease given the propensity of alcohol ingestion to decrease insulin-mediated glucose uptake in addition to chronic pancreatic damage [5,6].

Pharmacological management of DM in patients with chronic liver disease is still controversial. Due to increased risk of lactic acidosis, metformin is not recommended as a first-line treatment for DM in patients with cirrhosis [2]. Insulin secretagogues such as SUs, which are used as second-line treatment of DM in the general population, are generally considered safe in patients with liver disease, although issues regarding their efficacy have been raised in the setting of alcoholic liver disease and pancreatic damage [2]. In particular, patients with decompensated cirrhosis are more prone to hypoglycemia and have shown a decreased capacity to counteract hypoglycemia secondary to these medications [2,7]. Since SUs are metabolized by the liver, a decrease in breakdown of these medications in the setting of liver cirrhosis may result in increased plasma concentrations, thereby increasing the risk of hypoglycemia [7].

Additionally, hypoalbuminemia in the setting of chronic liver disease may result in reduced protein-binding of SUs, which may also result in increased SU plasma concentrations, thereby increasing the risk of hypoglycemia [3,8]. In the setting of alcohol consumption, decreased potency of SUs may result from alcohol-induced enzymatic degradation of SUs in addition to damage of beta islet cells of the pancreas [9,10]. Despite this decreased potency, patients taking SUs who have not abstained from alcohol are at an increased risk of hypoglycemia due to the inhibitory effects of alcohol on hepatic gluconeogenesis [7]. Another contributory factor to hypoglycemia is the increased prevalence of malnutrition among patients with chronic liver disease and patients with chronic alcohol use [3,7].

Given the limited studies evaluating the pharmacokinetics and safety of oral anti-diabetic drugs in patients with cirrhosis, the use of SUs remains a controversial issue. The American Diabetes Association (ADA) and the European Association for the Study of Diabetes (EASD) stated that it is “unwise to use sulfonylureas, particularly glyburide” in the setting of severe hepatic disease due to the risk of hypoglycemia [11]. Khan et al. reported the dosage of SUs should be decreased to half of the usual dosage in patients with cirrhosis, particularly if they have not abstained from alcohol [12]. Scheen et al. explained that SUs can be given with close monitoring for hypoglycemia [7]. Gangopadhyay and Singh reported that while SUs should be avoided in patients with cirrhosis, lower doses may be considered in patients with Child-Pugh Class A and B and should be completely avoided in patients with Child-Pugh Class C [3].

The patient described in this case report had a longstanding history of alcoholic cirrhosis, was still drinking alcohol intermittently, and was on glyburide. He developed hypoglycemia after stopping drinking alcoholic beverages for three days, which may have allowed glipizide to be more potent since its alcohol-induced enzymatic degradation was reduced. Patients with medication non-compliance, alcoholism, malnutrition, and recurrent hospital admissions have unpredictable outcomes regardless of whether their liver disease is decompensated or stable. Given the high risk of death and long-term neurological damage from hypoglycemia, we believe that SUs should be contraindicated in patients with chronic liver disease.

## Conclusions

The risk of hypoglycemia secondary to SUs in patients with cirrhosis is more likely to be higher than the general diabetic population due to associated behavior such as alcoholism, malnutrition, medication interaction, decreased hepatic clearance, and further decompensation. The use of SUs in this patient population should be contraindicated to avoid further decompensation and death following hypoglycemia.
